# Interferon-*γ*-Mediated Natural Killer Cell Activation by an Aqueous* Panax ginseng* Extract

**DOI:** 10.1155/2015/603198

**Published:** 2015-11-16

**Authors:** Kazuyoshi Takeda, Ko Okumura

**Affiliations:** ^1^Division of Cell Biology, Biomedical Research Center, Graduate School of Medicine, Juntendo University, Bunkyo-ku, Tokyo 113-8421, Japan; ^2^Department of Biofunctional Microbiota, Graduate School of Medicine, Juntendo University, Bunkyo-ku, Tokyo 113-8421, Japan; ^3^Atopy (Allergy) Research Center, Graduate School of Medicine, Juntendo University, Bunkyo-ku, Tokyo 113-8421, Japan; ^4^Department of Immunology, Juntendo University School of Medicine, Bunkyo-ku, Tokyo 113-8421, Japan

## Abstract

*Panax ginseng* extracts are used in traditional herbal medicines, particularly in eastern Asia, but their effect on natural killer (NK) cell activity is not completely understood. This study aimed to examine the effects of* P. ginseng* extracts on the cytotoxic activity of NK cells. We orally administered* P. ginseng* extracts or ginsenosides to wild-type (WT) C57BL/6 (B6) and BALB/c mice and to B6 mice deficient in either recombination activating gene 2 (RAG-2) or interferon-*γ* (IFN-*γ*). We then tested the cytotoxic activity of NK cells (of spleen and liver mononuclear cells) against NK-sensitive YAC-1 cells. Oral administration of* P. ginseng* aqueous extract augmented the cytotoxicity of NK cells in WT B6 and BALB/c mice and in RAG-2-deficient B6 mice, but not in IFN-*γ*-deficient B6 mice. This effect was only observed with the aqueous extract of* P. ginseng*. Interestingly, the ginsenosides Rb1 and Rg1 did not augment NK cell cytotoxicity. These results demonstrated that the aqueous* P. ginseng* extract augmented NK cell activation* in vivo* via an IFN-*γ*-dependent pathway.

## 1. Introduction

*Panax ginseng* (*P. ginseng*) roots provide a health food and are used to prepare a range of remedies employed in traditional Asian medicine.* P. ginseng* has been reported to possess several biological activities including antiaging, antidiabetic, anticarcinogenic, analgesic, antipyretic, antistress, antifatigue, and tranquilizing effects [1–3]. Ginsenosides, saponin molecules that are unique to* Panax* species, are generally considered to be the primary active pharmacological components of* P. ginseng* [4]. These compounds are also used as markers for the quality control and standardization of commercially available extracts of* Panax* species. In twenty different saponins, the ginsenosides, Rb1, Rg1, and Rd1, and the notoginsenoside, R1, are considered to be the major bioactive components [5]. In addition, nonsaponin compounds present in* P. ginseng* are also reported to have hypoglycemic [6] and antitumor activities [7].

There are a number of reports concerning the immunological effects of* P. ginseng*. However, it is unclear whether* P. ginseng* activates or suppresses immune responses. Some reports have shown that* P. ginseng* augments the activity of natural killer (NK) cells [8–13], which play an important role in innate immunity against infection and tumor development [14, 15]. However, the mechanisms involved in this augmentation of NK cell activity have not been identified. The present study examined the effects of* P. ginseng* on NK cell cytotoxicity using gene-targeted mice. We demonstrated that an aqueous extract of* P. ginseng* augmented the cytotoxicity of NK cell depending on interferon-*γ* (IFN-*γ*).

## 2. Materials and Methods

### 2.1. Mice

All mouse experiments were approved by Animal Care and Use Committee in Juntendo University (number 25008 on 2013/2/14 and number 260012 on 2014/2/7). Wild-type (WT) male C57BL/6 (B6) and BALB/c mice were purchased from Charles River Japan Inc. (Yokohama, Japan) at 6 weeks of age. IFN-*γ* deficient (IFN-*γ* ^−/−^) and recombination activating gene 2- (RAG-2-) deficient (RAG-2^−/−^) B6 mice were derived as described previously [16, 17]. All mice were maintained under specific pathogen-free conditions and used in accordance with the institutional guidelines of Juntendo University.

### 2.2. Reagents

**Figure 1:**
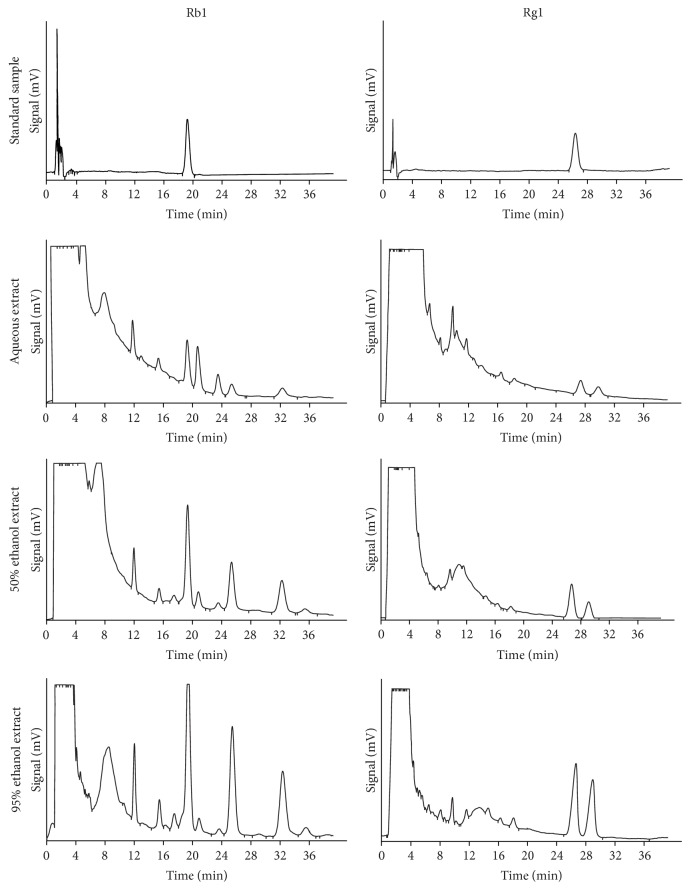
Concentration of Rb1 and Rg1 in aqueous extract, 50% ethanol extract, and 95% ethanol extract. Standard sample was diluted to 0.001%. Each sample was diluted to 0.2%. High-performance liquid chromatography (HPLC) was performed under the following conditions according to the Ginseng section of the revised 16th edition of the Japanese Pharmacopoeia: injected volume, 20 *μ*L each; detector, UV absorption spectrometer (203 nm); column, stainless, 150 × 4.6 mm; octadecyl-silica (ODS), 5 *μ*m. Mobile phase, column temperature, and flow rate for ginsenosides Rb1 and Rg1 were water/acetonitrile (4 : 1) and water/acetonitrile (7 : 3), 30 and 40 degrees Celsius, and retention time of about 25 and 20 minutes, respectively.

Aqueous (batch number 67EX0326) and ethanol (50% [batch number 67-X-282] or 95% [batch number 67-X-281]) extracts of* P. ginseng* were provided by Nagaoka & Co., Ltd. (Nishinomiya, Hyogo, Japan). Briefly, the aqueous extract was prepared with water at 85°C for 20 h, and the ethanol extracts were prepared by refluxing with 50% ethanol or 95% ethanol for 16 h. Then, extract solutions are concentrated by evaporation in vacuo to give the final extract products. The Rb1 and Rg1 levels in the extracts were estimated by liquid chromatography spectrometry using area normalization methods (Figure 1). Following the instructions of the Japanese Pharmacopoeia (http://jpdb.nihs.go.jp/jp16e/jp16e.pdf), we estimated the concentration of Rb1 and Rg1. Rb1 and Rg1 were purchased from Abcam Biochemicals (Cambridge, UK). Rb1 and Rg1 were mixed according to the constituents of several commercially available* P. ginseng* extracts. Extracts and ginsenosides were orally administered to mice as a suspension in distilled water (200 *μ*L).

### 2.3. Cytotoxicity Assay

Liver and spleen mononuclear cells (MNCs) were prepared as previously described [16–18]. The cytotoxic activity of MNCs was assessed against the NK cell-sensitive YAC-1 cells, using a standard ^51^Cr release assay [16–18]. Briefly, Na_2_^51^CrO_4_-labeled YAC-1 cells were coincubated with serial dilutions of MNCs on a round-bottom 96-well plate (Corning Inc., Corning, NY) in 5% CO_2_ in air at 37°C for 4 h. Spontaneous release was determined by incubating the target YAC-1 cells in medium, and maximal release was measured by placing the target cells in medium containing 1% Triton X-100. The supernatants were collected to measure radioactivity released during incubation, and the percentage of specific lysis was calculated according to the formula Specific lysis = (experimental release − spontaneous release)/(maximal release − spontaneous release) × 100. As the positive control of cytotoxic assay, we concurrently examined the NK cell activity after the intraperitoneal injection of IL-12 (eBioscience, San Diego, CA) and/or IL-28 (R&D Systems, Minneapolis, MN), which has been described to augment NK cell cytotoxic activity [18, 19], using the same target cells at the same cytotoxic assay condition.

### 2.4. Flow Cytometric Analysis

After preincubation with an anti-mouse CD16/32 (2.4G2) monoclonal antibody (mAb) to reduce nonspecific binding of mAbs to Fc*γ* receptors, cell surface molecules were stained with FITC-conjugated anti-mouse CD3 mAb (145-2C11) and phycoerythrin-conjugated anti-NK1.1 mAb (PK136) (for B6 mice) or anti-CD49b mAb (DX5) (for BALB/c mice) and analyzed by FACS Caliber (BD Bioscience, San Jose, CA) [18]. All reagents were purchased from eBioscience.

### 2.5. Statistical Analysis

Data were analyzed by a two-tailed Student's *t*-test. *P* values less than 0.05 were considered statistically significant.

## 3. Results

### 3.1. Augmentation of NK Cell Cytotoxic Activity following Oral Administration of Aqueous* P. ginseng* Extract

**Figure 2:**
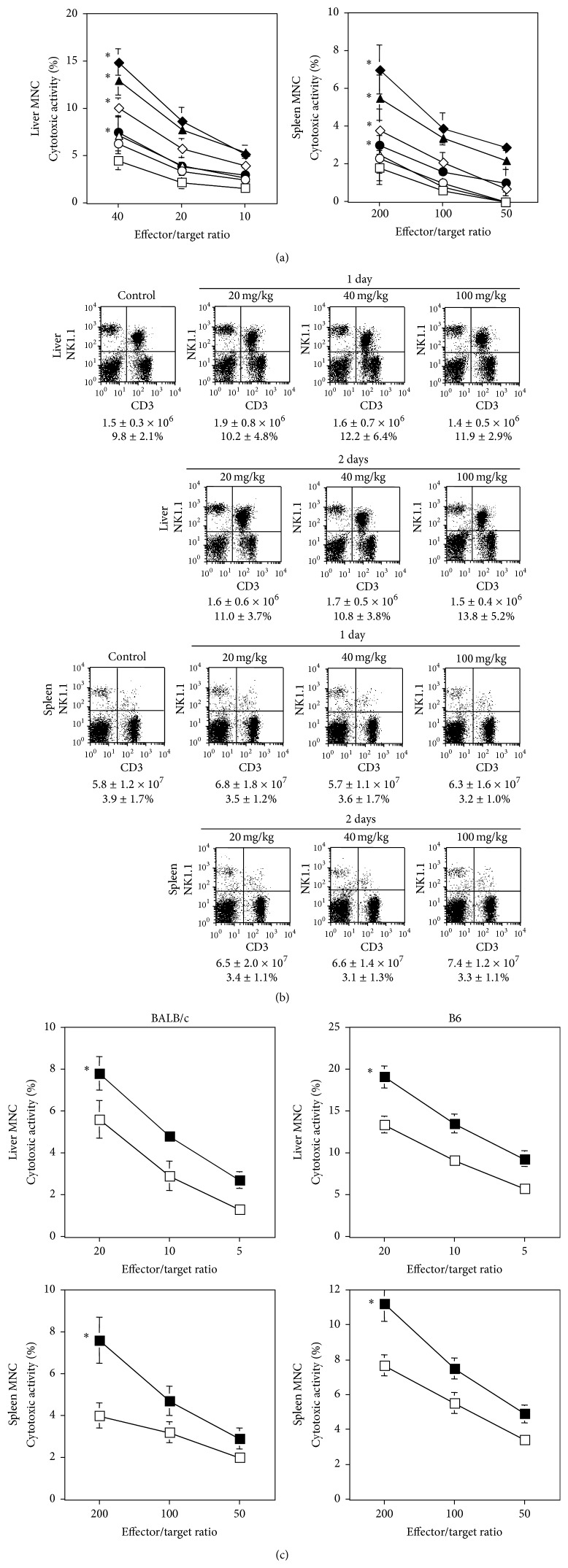
Activation of NK cytotoxicity by oral administration of aqueous* P. ginseng* extract. (a) WT B6 mice (*n* = 3 in each group) were administered with aqueous extract of* P. ginseng* (20 mg/kg; circle, 40 mg/kg; triangle, 100 mg/kg; diamond) on days -2 and -1 (closed symbols) or on day -1 (open symbols). Control mice (open square) (*n* = 3) were administered with the same volume (200 *μ*L) of water on days -2 and -1. Liver and spleen MNCs were prepared and cytotoxicity was analyzed using NK-sensitive YAC-1 cells, at the indicated effector/target ratios. Data are shown as the mean ± SD of triplicate samples of all tested mice. Similar results were obtained in three independent experiments. ^*∗*^ *P* < 0.05 as compared with the control at each effector/target ratio. (b) The populations of liver and spleen MNCs were analyzed by flow cytometry. MNC numbers and % of NK cells are indicated below every panel. Data are shown as mean ± SD of three mice in each group. Similar results were obtained in three independent experiments. (c) WT BALB/c and WT B6 mice (*n* = 3 in each group) were administered with aqueous extract of* P. ginseng* (50 mg/kg; closed square) or the same volume (200 *μ*L) of water (open square) on days -2 and -1. Liver and spleen MNCs were prepared and cytotoxicity was analyzed using YAC-1 cells at the indicated effector/target ratios. Data are shown as mean ± SD of triplicate samples of all tested mice. Similar results were obtained in three independent experiments. ^*∗*^ *P* < 0.05 as compared with the control at each effector/target ratio.

Oral administration of 20, 40, or 100 mg/kg aqueous* P. ginseng* extract to WT B6 mice for 2 days augmented the cytotoxicity of liver and spleen MNCs against YAC-1 cells (Figure 2(a)). Administration of 100 mg/kg aqueous* P. ginseng* extract for 1 day also significantly augmented the cytotoxic activity, although 1-day intake of 20 or 40 mg/kg of this extract did not augment cytotoxicity (Figure 2(a)). Neither the overall MNC numbers nor the NK cell populations in the liver and spleen significantly increased (Figure 2(b)). Oral administration of aqueous* P. ginseng* extract (50 mg/kg) augmented liver and spleen NK cell cytotoxicity in BALB/c and B6 mice, suggesting that there was no strain difference in this response (Figure 2(c)). None of the mice treated with oral aqueous* P. ginseng* extract showed signs of hepatotoxicity (assessed by measurement of serum alanine aminotransferase and aspartate aminotransferase) or systemic toxicity (assessed by observation of body weight, gross appearance, and behavior) (data not shown). These results showed that NK cytotoxicity, but not NK cell number, was increased by oral administration of aqueous* P. ginseng* extract.

### 3.2. Requirement for IFN-*γ*, but Not for Acquired Immune Cells, for* P. ginseng*-Induced NK Cell Activation* In Vivo*

**Figure 3:**
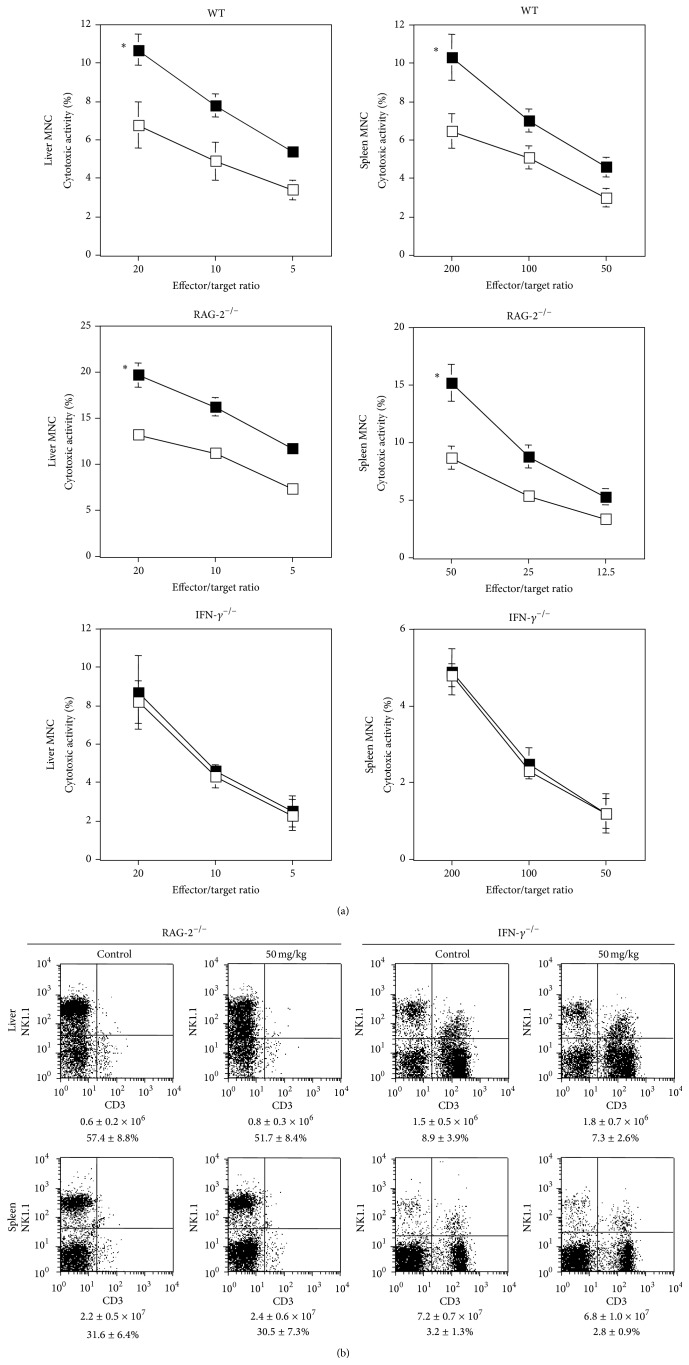
IFN-*γ*-dependent NK cell activation by oral administration of aqueous* P. ginseng* extract. (a) WT, RAG-2^−/−^, or IFN-*γ* ^−/−^ B6 mice (*n* = 3 in each group) were orally administered with aqueous* P. ginseng* extract (50 mg/kg; closed square) or the same volume (200 *μ*L) of water (open square) on days -2 and -1. Liver and spleen MNCs were prepared and cytotoxicity was analyzed using YAC-1 cells at the indicated effector/target ratios. Data are shown as mean ± SD of triplicate samples of all tested mice. Similar results were obtained in three independent experiments. ^*∗*^ *P* < 0.05 as compared with the control at all effector/target ratios. (b) The populations of liver and spleen MNCs were analyzed by flow cytometry. The MNC number and % of NK cells are indicated below every panel. Data are shown as mean ± SD of three mice in each group. Similar results were obtained in three independent experiments.

To investigate the contribution of acquired immune cells (T cells, NKT cells, and B cells) and IFN-*γ* on the observed* P. ginseng*-mediated augmentation of cytotoxicity* in vivo*, we orally administered aqueous* P. ginseng* extract (50 mg/kg) to RAG-2^−/−^ and IFN-*γ* ^−/−^ B6 mice. Liver and spleen MNCs showed augmented cytotoxicity when WT and RAG-2^−/−^ B6 mice received aqueous* P. ginseng* extract (Figure 3(a)). However, this treatment did not elevate the cytotoxicity of liver and spleen MNCs in IFN-*γ* ^−/−^ B6 mice (Figure 3(a)), indicating a critical role for IFN-*γ* in this effect. Neither MNC numbers nor the NK cell populations in the liver or spleen increased, even when cytotoxicity was significantly augmented in RAG-2^−/−^ B6 mice (Figure 3(b)), consistent with our observations in WT mice. These results indicated that oral administration of aqueous* P. ginseng* extract augmented NK cell cytotoxicity in an IFN-*γ*-dependent manner that was independent of acquired immune cell responses.

### 3.3. NK Cell Cytotoxicity Was Not Increased by Rb1 or Rg1

**Figure 4:**
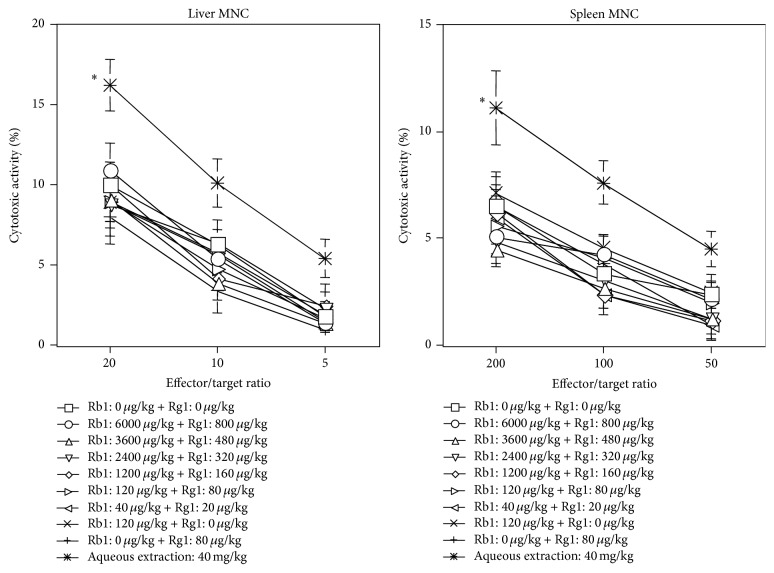
Activation of NK cytotoxicity by oral administration of aqueous* P. ginseng* extract, but not by administration of the ginsenosides, Rb1 and Rg1. WT B6 mice (*n* = 3 in each group) were administered with aqueous extract of* P. ginseng* (40 mg/kg), mixtures of the indicated amounts of Rb1 and/or Rg1, or the same volume (200 *μ*L) of water on days -2 and -1 as indicated. Liver and spleen MNCs were prepared and cytotoxicity was analyzed using YAC-1 cells at the indicated effector/target ratios. Data are shown as mean ± SD of triplicate samples of all tested mice. Similar results were obtained in three independent experiments. ^*∗*^ *P* < 0.05 compared with the control at all effector/target ratios.

Ginsenosides are the major constituents of* P. ginseng*, and Rb1 and Rg1 are generally considered to be the main effector saponins of the > 20 reported ginsenosides [5]. We examined whether Rb1 and/or Rg1 activated NK activity* in vivo*. WT B6 mice were orally administered with mixtures of Rb1 and Rg1 that were prepared following analysis of the constituents of several commercially available* P. ginseng* extracts (data not shown). Interestingly, cytotoxic activity was not augmented following oral administration of either Rb1 or Rg1 (Figure 4).

### 3.4. An Ethanol* P. ginseng* Extract Did Not Augment NK Cell Cytotoxicity

**Figure 5:**
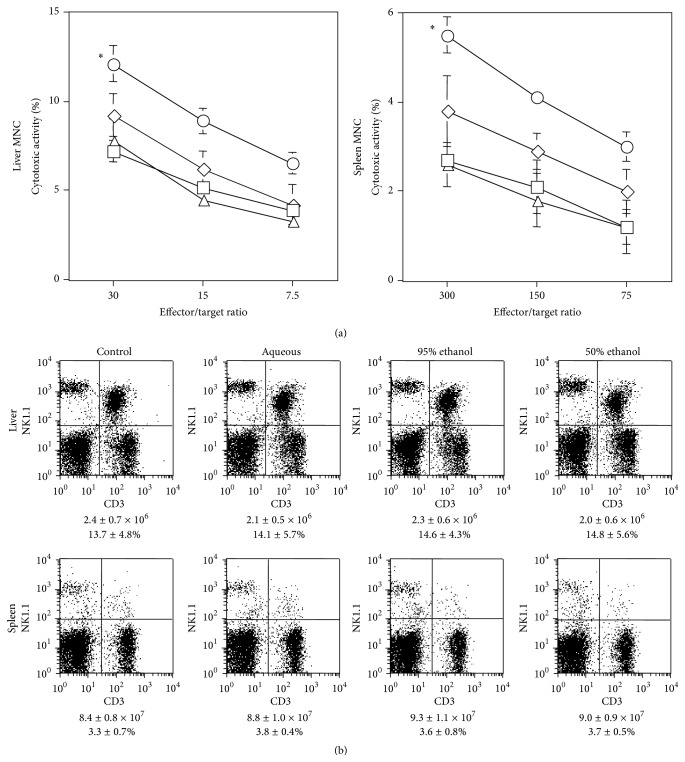
Activation of NK cytotoxicity by oral administration of aqueous, but not ethanol,* P. ginseng* extract. (a) WT B6 mice (*n* = 3 in each group) were administered with aqueous extract (circle), 95% (diamond) or 50% (triangle) ethanol extracts of* P. ginseng* (100 mg/kg), or the same volume (200 *μ*L) of water (square) on days -2 and -1. Liver and spleen MNCs were prepared and cytotoxicity was analyzed using YAC-1 cells at the indicated effector/target ratios. Data are shown as mean ± SD of triplicate samples of all tested mice. Similar results were obtained in three independent experiments. ^*∗*^ *P* < 0.05 compared with the control at all effector/target ratios. (b) The populations of liver and spleen MNCs were also analyzed by flow cytometry. The MNC number and % of NK cells are indicated below every panel. Data are shown as mean ± SD of three mice in each group. Similar results were obtained in three independent experiments.

Both alcohol and aqueous extracts of* P. ginseng* are used in complementary and alternative medicines. Our analyses demonstrated that Rb1 and Rg1 were present at higher levels in alcohol extracts (Table 1). Thus, we examined the effect of ethanol extracts of* P. ginseng* on NK cell cytotoxicity. Oral administration of the 95% ethanol* P. ginseng* extract to WT B6 did not augment liver or spleen NK cell activity (Figure 5(a)). The 50% ethanol extract appeared to increase cytotoxicity, but this effect was not statistically significant (Figure 5(a)). Ethanol extract influenced neither the number of MNCs nor the NK cell populations (Figure 5(b)). These results suggested that some constituent that was present at higher levels in the aqueous* P. ginseng* extract augmented NK cell activity* in vivo*.

## 4. Discussion

In this study, we explored the activation of NK cell cytotoxicity following oral administration of an aqueous* P. ginseng* extract to WT, RAG-2^−/−^, and IFN-*γ* ^−/−^ mice. In RAG-2^−/−^ mice, but not in IFN-*γ* ^−/−^ mice, oral consumption of this extract augmented NK cell cytotoxicity to the same extent as that observed in WT mice. Administration of Rb1, Rg1, or an ethanol extract of* P. ginseng* did not augment NK cell activity. None of the treatments studied affected the MNC or NK cell numbers, even when NK cytotoxicity was augmented. This is the first report to use gene-targeted mice to demonstrate that an aqueous* P. ginseng* extract augmented NK cell cytotoxicity in an IFN-*γ*-dependent manner.

Extracts of* P. ginseng* are orally ingested as one of the most common complementary medicines for cancers and other diseases, particularly in eastern Asia. The present study therefore used a mouse oral administration model to examine the mechanisms involved in the effects of* P. ginseng* on NK cell activity.* P. ginseng* was reported to augment NK cell activity in rodents and humans [9–13]. Interestingly, consistent with our observations, synergistic increases in the level of serum interleukin- (IL-) 12 and IFN-*γ* accompanied by NK cell activation [10] and NK cell activation with no substantial effect on T or B cell responses [11] were reported. We hypothesize that* P. ginseng* augments NK cell activity via similar mechanisms in humans and mice.

Epidemiological studies demonstrated an association between ginseng intake and decreased cancer incidence and growth [20, 21]. Administration of* P. ginseng* has also been reported to inhibit tumor development and/or metastasis, as well as NK cell activation, in mice [9, 22]. The present study demonstrated that oral administration of an aqueous* P. ginseng* extract augmented NK cell cytotoxicity through a mechanism involving IFN-*γ*. NK cell cytotoxicity and IFN-*γ* are generally regarded as critical effectors of immune surveillance against tumors [15, 23–25]. It was also reported that* P. ginseng* induced T helper 1 (Th1) and macrophage cytokines, generating lymphokine-activated killer cells in synergy with IL-2 [26]. Thus, cytotoxic immune cells would contribute to the antitumor effects of* P. ginseng*. In addition, ginseng saponins (such as Rg1) have been reported to inhibit the production of inflammatory cytokines, TNF-*α* and IL-6, [27–30], which are critical for inflammation-related tumor development [31]. Therefore, the antitumor effects of* P. ginseng* would be mediated by both the activation of immune surveillance and inhibition of inflammation-related tumor development.

The results of the present study suggested that some constituent of the aqueous extract, but not of the ethanol extract, augmented NK cell cytotoxicity. Some previous reports have demonstrated that* P. ginseng* had immunosuppressant effects [30, 32, 33]. It was previously reported that monocyte function, monocyte differentiation into dendritic cells, and toll-like receptor-mediated dendritic cell activation were attenuated by* P. ginseng* [30, 33]. Notably, alcohol extracts of* P. ginseng* that contained high levels of the ginsenosides, Rb1 and Rg1, were used in these reports. An acidic polysaccharide from* P. ginseng* was reported to induce regulatory T cells and ameliorate autoimmune disease [34]. It was reported that two different* P. ginseng* extracts had different immunological effects [12]. The effects of various* P. ginseng* extracts probably differ due to their different levels of active compounds.

A variety of receptors, such as toll-like receptors and C-type lectin receptors, have been identified on macrophages, dendritic cells, and innate immune cells [35, 36]. These receptors are thought to mediate sensitivity to microbes, probiotics, and complementary and alternative medicines [37, 38]. The IFN-*γ*-dependent and acquired immune cell-independent NK cell activation observed following* P. ginseng* administration is fairly similar to that associated with probiotics and *β*-glucan [17, 39], indicating that toll-like receptors and/or C-type lectin receptors may mediate the effects of* P. ginseng* on NK cell activation. Moreover, ginsenosides are metabolized by colonic bacteria, and these active metabolites mediate their immune and anticancer effects [18]. Taken together, the range of compounds present in* P. ginseng* extracts, the individual differences in the expression profiles of innate immune receptors in human subjects, and the intestinal bacterial flora would all influence the biological effects of* P. ginseng* in humans. To improve the therapeutic benefits of* P. ginseng*, further studies are required to elucidate the complex molecular mechanisms involved in the biological effects of* P. ginseng*.

## Figures and Tables

**Table 1:** Rb1 and Rg1 concentrations in the *P. ginseng* extracts.

	Rb1 (%)	Rg1 (%)
Aqueous extract	0.52	0.04
95% ethanol extract	2.66	1.09
50% ethanol extract	1.23	0.50

## Abbreviations

IFN: Interferon
MNCs: Mononuclear cells
NK: Natural killer
RAG-2: Recombination activating gene 2
WT: Wild-type
IL: Interleukin
SD: Standard deviation.
